# Family-Centered Rehabilitation and Parenting Resilience in the Care of an Infant Born at 22 Weeks: A Case Study

**DOI:** 10.7759/cureus.89567

**Published:** 2025-08-07

**Authors:** Yusuke Aoyama, Tomohiro Mori, Masako Nagata, Yoshiaki Sato

**Affiliations:** 1 Department of Rehabilitation, Nagoya University Hospital, Nagoya, JPN; 2 Department of Rehabilitation, Nagoya University Graduate School of Medicine, Nagoya, JPN; 3 Department of Psychology and Human Developmental Sciences, Psychological Support and Research Center for Human Development, Nagoya University, Nagoya, JPN; 4 Division of Neonatology, Center for Maternal-Neonatal Care, Nagoya University Hospital, Nagoya, JPN

**Keywords:** case study, development, extremely preterm infant, family-centered care, occupational therapy, parenting resilience, self-regulation

## Abstract

This case report describes the implementation of Family-Centered Care (FCC) and developmental occupational therapy (OT) for an extremely preterm infant born at 22 weeks and one day of gestation, weighing 448 g. The infant experienced multiple complications, including necrotizing enterocolitis, sepsis, intraventricular hemorrhage, and respiratory distress, requiring prolonged intensive care. Due to physiological fragility and immature neurobehavior, a structured rehabilitation approach was introduced, integrating OT and caregiver participation based on FCC principles. The intervention aimed to enhance the infant’s self-regulation and support parenting resilience. Self-regulation was evaluated using the Brazelton Neonatal Behavioral Assessment Scale (NBAS), and parental resilience was assessed with the Parenting Resilience Elements Questionnaire (PREQ). The program was divided into three phases aligned with developmental stages and caregiver involvement. NBAS self-regulation scores improved from a median of 1 (low level) to 6 (ordinary level). PREQ scores for both parents also increased, reflecting stronger emotional bonding, better understanding of the infant’s cues, and improved confidence in caregiving. Caregivers were gradually engaged in routines such as suckling, bathing, and developmental play. Collaborative role-sharing among occupational therapists, physical therapists, and nursing staff further supported the intervention. This case highlights how early FCC and developmental OT may promote neurodevelopment and psychological adaptation in families of extremely preterm infants.

## Introduction

Extremely preterm infants (EPI) born before 28 weeks face major physical challenges, while their families experience significant psychological, physical, and social burdens. Although neonatal intensive care has improved survival, long-term neurodevelopmental outcomes remain uncertain, necessitating ongoing family support [1-4]. Developmental support and rehabilitation are crucial in promoting motor, sensory, and cognitive development in preterm infants [5-7]. In recent years, the importance of family-centered approaches has become more widely recognized, resulting in the implementation of Family-Centered Care (FCC) and Family-Integrated Care (FICare). These elements contribute to improved developmental outcomes for infants, enhanced parent-child relationships, and overall family functioning [8]. A systematic review by Hodgson et al. (2025) reported that the FCC contributes to increased weight gain, shorter hospital stays, and higher breastfeeding rates in preterm infants, as well as reduced parental stress and greater satisfaction [9]. Similarly, a systematic review by Ding et al. (2023) demonstrated that the FICare provides comparable benefits, including enhanced infant weight gain, shorter hospitalization, improved breastfeeding outcomes, and positive effects on parental stress and satisfaction [10]. Furthermore, developmental support programs, including occupational therapy, have been reported to facilitate sensory-motor integration and emotional regulation, while also influencing parent-infant interactions [11]. A single-center randomized controlled trial (RCT) by Fucile et al. (2024) suggested that parent-delivered sensorimotor interventions for preterm infants are effective in promoting infant developmental outcomes and enhancing parental caregiving behaviors [12].

Recently, the concept of parenting resilience has received growing attention. Parenting resilience refers to a caregiver's ability to maintain psychological stability and positive engagement with their child, even when faced with adversity such as a child's disability or caregiving difficulties. Emerging research supports the notion that the FCC and professional support play an important role in fostering this resilience [13].

However, there is a lack of practical reports integrating the FCC with developmental rehabilitation in settings such as the Neonatal Intensive Care Unit (NICU) and Growing Care Unit (GCU), particularly those addressing neurobehavioral development in infants and family psychological adaptation. In particular, reports on infants born at extremely early gestational ages, such as around 22 weeks, are extremely limited [14,15]. Further clinical practice development and research are needed in this area. Here, we report a case of an extremely preterm and extremely low birth weight infant, born at 22 weeks and 1 day of gestation with a birth weight of 448 grams, who received integrated FCC and developmental rehabilitation.

## Case presentation

Present illness

At the time of this writing, the patient was a 235-day-old (55 weeks and 5 days postmenstrual age (PMA)) female infant who was born at 22 weeks and 1 day of gestation with a birth weight of 448 g. Her comorbidities included necrotizing enterocolitis (NEC), sepsis, neonatal respiratory distress syndrome, intraventricular hemorrhage, osteopenia of prematurity, and retinopathy of prematurity. The infant was delivered via an emergency cesarean section at a different medical facility. Immediately following the birth, intubation was initiated to manage the infant's respiratory status. Apgar scores were six at both one and five minutes. On Day 18, an abdominal X-ray revealed free air, and the patient was subsequently transferred to our hospital with septic shock secondary to NEC. On the same day, an emergency stoma creation and bowel resection were performed. Thereafter, a recurrence of NEC was identified on Day 31, necessitating a secondary bowel resection. Although she was extubated on Day 70, she sustained fractures in her limbs due to malnutrition on Day 96. She was weaned off the ventilator on Day 107 and underwent stoma closure and a third bowel resection on Day 157.

Initial assessment (47 + 6 weeks PMA)

Physical therapy (PT) was initiated on Day 157, and occupational therapy (OT) was initiated on Day 181. PT addressed motor development and worked collaboratively with OT on posture and positioning adjustments to support the infant's physical and sensory needs. At the time of the initial intervention, the infant’s length was 41.5 cm (−7.5 SD) and body weight was 1830 g (−5.5 SD). Self-regulation was assessed using seven items related to self-regulatory function, derived and adapted from the Neonatal Behavioral Assessment Scale (NBAS), version 4 [16-18]. The NBAS was administered by a staff member licensed to perform the assessment. The median score across all domains was 1, indicating a classification of Low Level of Self-Regulation (LLSR). From a sensory perspective, gazing and tracking were absent. Nutritional intake was provided via tube feeding, with a daily volume of 280 ml. Although parent-infant bonding was observed, the parents’ involvement in daily caregiving activities, such as bathing and holding, remained limited. Parenting resilience was assessed using the Parenting Resilience Elements Questionnaire (PREQ), with total scores of 67 for the mother and 66 for the father [19]. These assessments indicated that the infant exhibited underdeveloped self-regulation abilities. Furthermore, the family’s level of parenting resilience appeared insufficient. Parent-infant bonding was insufficiently established with both the mother and the father. In response, nursing and rehabilitation staff collaborated to actively implement the FCC and develop a plan for developmental rehabilitation.

OT intervention

The intervention for this case was designed based on the principles of the FCC, with three core objectives: ensuring the infant’s physiological stability, promoting family participation in care, and enhancing parenting resilience. OT sessions were held 3 times per week, lasting 20-40 minutes each. Direct interventions for the infant focused on improving self-regulation through positioning and multisensory play. To strengthen child care resilience, interventions corresponding to the PREQ assessment domains were used. To enhance parental understanding of the child’s characteristics and promote a positive perception, we provided direct explanations regarding the infant’s physiological responses and behavioral patterns during parental visits. In order to raise awareness of social support, both parents were encouraged to attend visits together. During these visits, they received direct guidance. Staff roles were clarified to ensure that parents knew whom to contact at any time. Nurses were responsible for overall caregiving, including suckling; physical therapists addressed motor development; and occupational therapists focused on sensory stimulation and play. OT and PT also collaborated on posture and positioning adjustments. There were no clear distinctions between OT and PT regarding posture and positioning adjustments. The therapists had a shared understanding and collaborated to explore positioning strategies that could promote self-regulation and encourage play. These strategies were regularly discussed and shared with the nursing staff.

Evaluation methods

Self-Regulation in the NBAS

The NBAS is a tool used to evaluate neonatal behavioral capacities and adaptive functioning [16]. This tool assesses six domains: Habituation, Orientation, Motor System, State Regulation, Autonomic Nervous System, and Reflexes. The behavioral items (excluding Reflexes) are rated on a nine-point Likert scale. Self-regulation is evaluated using seven behavioral items: peak of excitement, rapidity of build-up, irritability, lability of states, cuddliness, consolability, and self-quieting. Based on the median score of these seven items, self-regulation is categorized as follows: 7-9 indicates a High Level of Self-Regulation (HLSR), 4-6 indicates an Ordinary Level of Self-Regulation (OLSR), and 1-3 indicates a Low Level of Self-Regulation (LLSR) [17,18].

The PREQ

The PREQ is a self-administered questionnaire developed to assess parental resilience in raising children with developmental disorders [19]. It consists of three domains: knowledge of the child’s characteristics, perception of social support, and positive perception of parenting. The PREQ has demonstrated reliability and validity, and higher scores indicate greater parenting resilience.

Intervention process

Early Phase of the FCC (48+1 Weeks PMA to 50+1 Weeks PMA)

At the beginning of the OT intervention, the NBAS had a median self-regulation capacity of one and LLSR status. The infant exhibited behaviors, such as arching during crying and difficulty with self-regulation, which made it challenging for the family to engage in daily care. As a result, the FCC phased in the system. In the initial phase, the parents were encouraged to assist in tube feeding management and observation during daily care. During rehabilitation sessions, their involvement began with observing and supporting positioning techniques and methods to promote self-regulation. The rehabilitation team consisted of OT and PT. PT addressed motor development, while OT focused on sensory stimulation and play. OT and PT also collaborated on posture and positioning adjustments. OT interventions focused on promoting self-regulation through positioning and multisensory play. Specifically, the infant's body was surrounded with urethane foam to increase the surface contact area, and additional pressure stimulation was applied using towels. Oral stimulation included the use of an empty pacifier and gentle tapping on the body to provide multisensory input. Furthermore, both the therapist and family members applied gentle pressure to the palms and soles, while vestibular input was provided by gentle rocking using a baby chair.

Middle Phase of the FCC (50+2 Weeks PMA to 52+5 Weeks PMA)

 The median self-regulatory ability of the NBAS was four and OLSR status, so the PREQ was re-evaluated. The PREQ was re-administered, with the mother scoring 92 and the father 78. The FCC at this stage included more practical involvement in caregiving tasks such as dressing, diaper changing, and supervised bathing. Rehabilitation activities expanded to include multisensory play using visual and tactile elements. OT interventions for the infant included high-contrast toys that produced sounds to stimulate both vision and hearing. The family and therapist guided the infant’s visual tracking by moving toys at a distance of approximately 30 cm. Additionally, exercises were introduced to support head control.

Final Phase of the FCC (52+6 Weeks PMA to 55 Weeks PMA)

**Figure 1:**
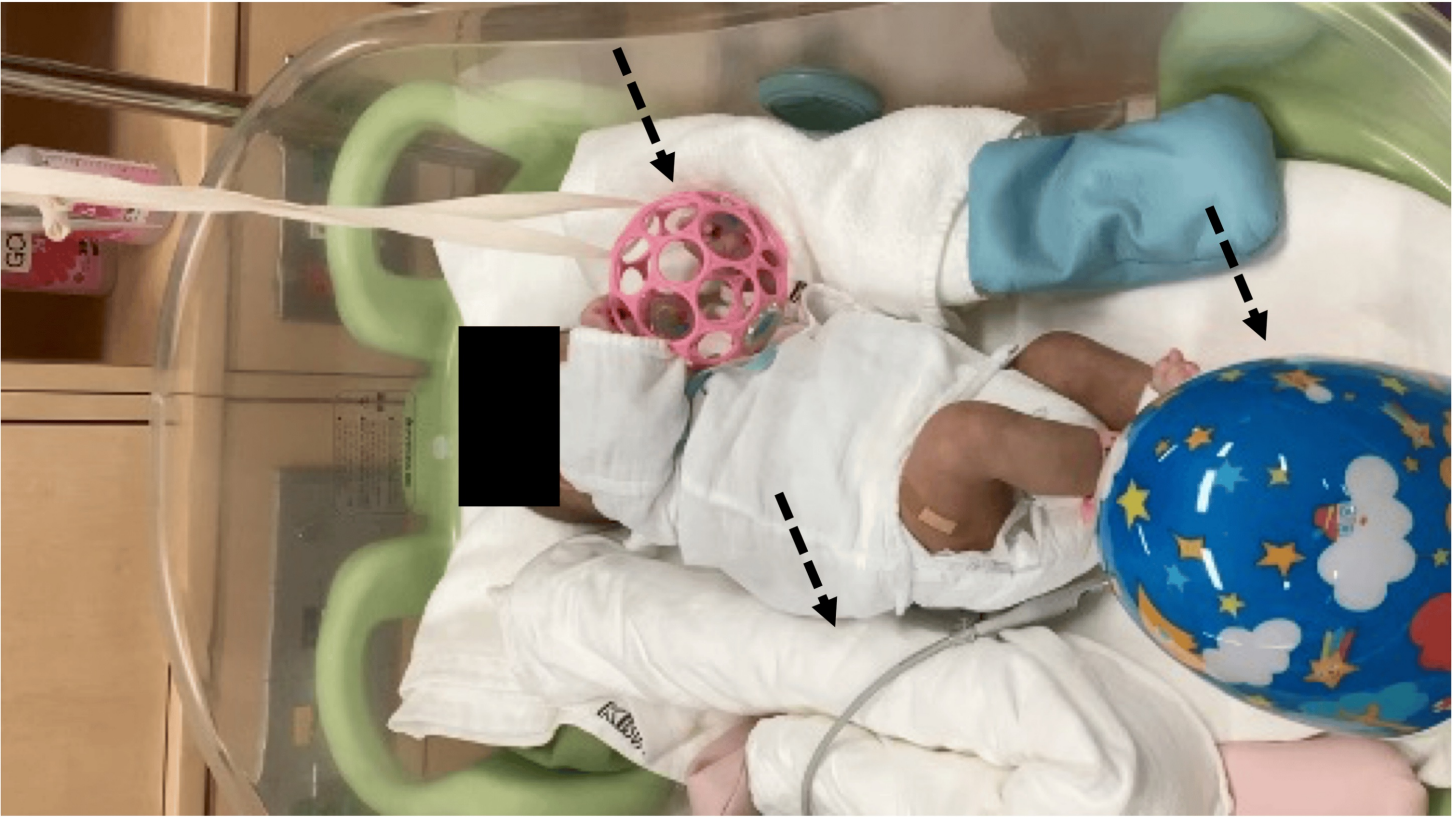
Adjustment of the environment on the bed at the time of the final evaluation To avert the occurrence of an arching back, towels were strategically positioned at the back. Toys that were easy to grasp were placed in the setting. In addition, toys that produced sounds when kicked were placed beneath the feet of the participants. The purpose of this was to integrate somatosensory and lower limb movements.

The median self-regulatory ability of the NBAS was 5, and the total PREQ score was 96 for the mother and 91 for the father. The FCC aimed to obtain more independent child-rearing behaviors, such as bathing and oral intake, for routine care. In rehabilitation, her family engaged in activities such as repositioning to prone and side, and playing with toys. In addition, the occupational therapist adjusted the bed environment to promote sensory integration of visual information and upper limb function (Figure 1). Before discharge from the hospital, the mother and child shared a room three times, and opportunities were provided for them to play together to promote development through daily care, with the parents sharing their observations and concerns at each session.

Final assessment (55 weeks PMA)

At the final assessment, the infant's self-regulation showed marked improvement, with the median score across all relevant NBAS domains reaching six. Simultaneously, parenting resilience had strengthened, with both parents scoring 94 on the PREQ (Table 1). These improvements reflected an enhanced understanding of the infant's behaviors and needs, which, in turn, facilitated more effective implementation of the FCC. Nutritional intake also increased to 660 ml/day (197 ml/kg) through oral feeding.

**Table 1:** Changes in evaluation scores and intervention content across FCC phases * The total score was 112 points. Abbreviations: FCC, Family-Centered Care; NBAS, Neonatal Behavioral Assessment Scale; LLSR, Low Level of Self-Regulation; OLSR, Ordinary Level of Self-Regulation; PREQ, Parenting Resilience Elements Questionnaire

Phase (Days)	NBAS Self-Regulation Score (Median)	PREQ Total Score (Mother)	PREQ Total Score (Father)	Understanding Child (Mother)	Understanding Child (Father)	Social Support (Mother)	Social Support (Father)	Positive Parenting (Mother)	Positive Parenting (Father)
Initial/Early Phase (Day 183-197)	1 (LLSR)	67	66	20	21	22	21	25	24
Middle Phase (Day 198-215)	4 (OLSR)	92	78	30	27	30	26	32	25
Final Phase (Day 216-231)	5 (OLSR)	96	91	33	30	32	30	31	31
Final Assessment (Day 231)	6 (OLSR)	94	94	32	32	30	31	32	31

## Discussion

This case involved an extremely preterm and extremely low birth weight infant, born at 22 weeks and 1 day of gestation with a birth weight of 448 g, who required extensive medical care due to multiple complications. In addition to marked immaturity in self-regulatory capacity, the infant’s family also exhibited a low level of parenting resilience. The implementation of rehabilitation based on the FCC was considered to have contributed to both the infant’s developmental progress and the enhancement of the family’s parenting resilience. Through the stepwise implementation of the FCC and developmental rehabilitation, the infant’s self-regulatory capacity, as assessed by the NBAS, improved from an LLSR to an OLSR. Self-regulation is recognized as an important indicator of neurobehavioral maturity in early infancy and plays a key role in social development, emotional regulation, and adaptation to developmental tasks [17,20]. In this case, the observed improvement in self-regulation suggests that the gradual sensory-motor interventions combined with stable parental engagement were effective in facilitating self-regulatory development [11].

Additionally, an improvement in parenting resilience was observed through the intervention provided in this case. According to Suzuki et al. (2015) [19], ongoing explanations from professionals and opportunities for practical participation in care are important factors that support caregivers’ psychological adaptation. The FCC not only promotes active parental involvement in childcare but also generates synergistic effects on children’s development [8,21,22]. These findings suggest that occupational therapy incorporating FCC principles and sensory-behavioral developmental support may be effective in enhancing both the infant’s self-regulation and the family’s parenting resilience, even in EPI with multiple complications. Given the extremely early gestational age of 22 weeks, there are still few reports both in Japan and abroad that address comprehensive developmental and family-centered support in such cases. This case provides valuable insights into the potential benefits and challenges of implementing interdisciplinary, developmentally supportive care for EPI.

One limitation of this case is the difficulty in establishing a standard pre-intervention baseline, due to COVID-19 visitation restrictions and the infant’s postoperative condition. Therefore, the initial OT evaluation was used as a reference point. To assess longitudinal change, two validated tools, the NBAS and the PREQ, were used. Patient performance using both tools showed improvement, which supports the effectiveness of FCC and developmental OT. The lower PREQ scores observed in the father may have been due to differences in hospital visit frequency between parents, particularly fewer opportunities for direct engagement by the father. This highlights the need for more equitable support strategies in future cases.

This case also emphasizes the importance of interdisciplinary collaboration in providing individualized and developmentally appropriate care for extremely preterm infants. The occupational therapist focused on sensory stimulation and emotional regulation. The physical therapist addressed motor development and posture. The nursing staff ensured physiological stability and feeding. These roles, grounded in each profession’s scope of practice, were integrated through clear communication and collaborative planning. Such coordinated efforts supported both neurodevelopmental outcomes and parental adaptation. Defining and integrating professional roles was essential for effective FCC. This is true in the NICU and GCU settings.

## Conclusions

This case study suggests that family-centered rehabilitation support for very preterm infants can have a positive impact on the child's development and the family's parenting resilience. This report is based on a single case and has limitations for generalization. However, this report may help clarify the importance of family support in the care of very preterm infants and the challenges in practice.
